# Mucocèle ethmoïdo-frontale survenant chez un diabétique de type 2

**DOI:** 10.11604/pamj.2015.20.431.6211

**Published:** 2015-04-29

**Authors:** Mahfoudhi Madiha, Khamassi Khaled

**Affiliations:** 1 Service de Médecine Interne A, Hôpital Charles Nicolle, Tunis, Tunisie; 2 Service ORL, Hôpital Charles Nicolle, Tunis, Tunisie

**Keywords:** Cellulite orbitaire, mucocèle, diabète, orbital cellulitis, mucocele, diabetes

## Image en medicine

Les mucocèles sont des formations pseudo-kystiques expansives des sinus de la face, dont la paroi est formée par une muqueuse sinusienne modifiée. Malgré leur bénignité, les mucocèles peuvent être une source de complications graves. Devant un aspect de cellulite orbitaire pouvant se manifester par une exophtalmie aiguë inflammatoire avec paralysie oculomotrice, voire même une ophtalmoplégie, le diagnostic de mucocèle surinfectée rompue dans l'orbite doit être évoqué, surtout s'il s'agit d'un terrain de diabète. L'imagerie permet de confirmer le diagnostic et le drainage en urgence s'impose afin de préserver le pronostic visuel. Patient âgé de 42 ans, diabétique de type 2, a consulté pour un œdème palpébral droit, une exophtalmie douloureuse et une rougeur périorbitaire évoluant depuis 2 jours. L'examen physique a objectivé une fièvre à 38,5°C, une exophtalmie droite, un ptosis et un œdème inflammatoire palpébral droit. La muqueuse nasale était congestive avec des méats moyens libres. Le patient a été alors hospitalisé pour cellulite orbitaire. La tomodensitométrie du massif facial a révélé un aspect de pyomucocèle ethmoïdo-frontale droite avec extension orbitaire. Le traitement a associé une antibiothérapie par voie parentérale, une équilibration du diabète et une marsupialisation de la mucocèle par voie endonasale. L’évolution était favorable avec disparition totale des signes ophtalmologiques. Aucune récidive n'a été notée après un recul de 2 ans.

**Figure 1:**
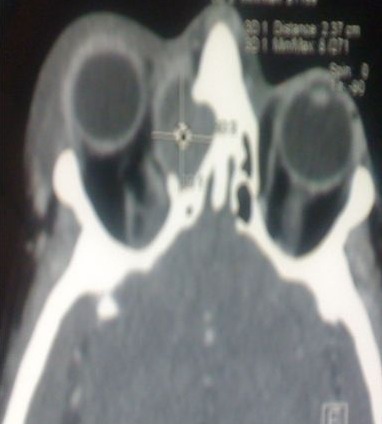
TDM du massif facial (coupe axiale): mucocèle ethmoïdo-frontale droite avec extension orbitaire

